# Adenoviruses in Lymphocytes of the Human Gastro-Intestinal Tract

**DOI:** 10.1371/journal.pone.0024859

**Published:** 2011-09-30

**Authors:** Soumitra Roy, Roberto Calcedo, Angelica Medina-Jaszek, Martin Keough, Hui Peng, James M. Wilson

**Affiliations:** Gene Therapy Program, Department of Pathology and Laboratory Medicine, Division of Transfusion Medicine, University of Pennsylvania, Philadelphia, Pennsylvania, United States of America; French National Centre for Scientific Research, France

## Abstract

**Objective:**

Persistent adenoviral shedding in stools is known to occur past convalescence following acute adenoviral infections. We wished to establish the frequency with which adenoviruses may colonize the gut in normal human subjects.

**Methods:**

The presence of adenoviral DNA in intestinal specimens obtained at surgery or autopsy was tested using a nested PCR method. The amplified adenoviral DNA sequences were compared to each other and to known adenoviral species. Lamina propria lymphocytes (LPLs) were isolated from the specimens and the adenoviral copy numbers in the CD4+ and CD8+ fractions were determined by quantitative PCR. Adenoviral gene expression was tested by amplification of adenoviral mRNA.

**Results:**

Intestinal tissue from 21 of 58 donors and LPLs from 21 of 24 donors were positive for the presence of adenoviral DNA. The majority of the sequences could be assigned to adenoviral species E, although species B and C sequences were also common. Multiple sequences were often present in the same sample. Forty-one non-identical sequences were identified from 39 different tissue donors. Quantitative PCR for adenoviral DNA in CD4+ and CD8+ fractions of LPLs showed adenoviral DNA to be present in both cell types and ranged from a few hundred to several million copies per million cells on average. Active adenoviral gene expression as evidenced by the presence of adenoviral messenger RNA in intestinal lymphocytes was demonstrated in 9 of the 11 donors tested.

**Conclusion:**

Adenoviral DNA is highly prevalent in lymphocytes from the gastro-intestinal tract indicating that adenoviruses may be part of the normal gut flora.

## Introduction

It has long been recognized that adenoviruses can establish latent infections in cells in organs such as the adenoids and tonsils. An adenoviral infectious episode typically resolves with the gradual reduction of the infectious viruses that can be detected by standard virological methods as was initially documented by two prospective studies [1], [2]; prolonged excretion of virus lasting for years was consistently observed in a minority of cases. However, it has also been known that even in the absence of active disease, viral dormancy can be demonstrated frequently by prolonged culture of tissue explants [3], [4], [5], [6]. In fact, the presence of adenoviral DNA has often been demonstrated in adenoidal or tonsillar explants [7] or T cells [8] in the absence of recoverable virus. Evidence for the presence of latent adenovirus in these tissues that could be re-activated by mitogenic stimulation of the T cells has recently been shown [9]. Furthermore, life-threatening adenoviral infections are a recognized complication following transplantation and are thought to be a consequence of immunosuppression induced by drugs that are routinely used to reduce the chance of transplant rejection, and are likely to result from the opportunistic replication of adenoviruses already present in the patient. However the location of such repositories other than the oropharyngeal immune organs has been hard to document; circulating lymphocytes in normal subjects have not yielded detectable adenoviral DNA by PCR-based methods [10], [11], but as mentioned above, such DNA is easily recovered from patients undergoing immunosuppressive therapy or in HIV-infected patients [10], [12], [13]. It is interesting to note that although adenoids or tonsils yield species C adenoviruses almost exclusively [9], species additionally identified in immunosuppressed individuals include serotypes belonging to the other common adenoviral species such as B, E, A and D; HIV patients appear to be especially susceptible to infections of the gastrointestinal tract by species D adenoviruses [13].

The question of adenoviral persistence in normal humans and the possible immunological consequences has recently come to the fore because of the hypotheses that have been proposed to account for the apparent trend towards increased acquisition of HIV in a vaccine trial (STEP) utilizing a replication-defective recombinant adenoviral vector [14], [15], [16]. This trend was most pronounced in the subset of individuals who harbored higher levels of neutralizing antibodies to the vector (presumably due to prior exposure to the virus). One favored explanation is that the vaccination of these individuals with the adenovirus vector resulted in an expansion of the adenovirus-reactive CD4+ T cells population, including those in the mucosa of the rectum, resulting in a more favorable milieu for HIV acquisition following rectal intercourse [17]. The submucosa of the lower gut is a very large immunological organ, and it is certainly plausible that a systemic response to an immune challenge, such as an anamnestic T cell response, may result in increased numbers of such cells in the gut mucosa.

Because adenoviral vectors are now being considered for widespread use in gene therapy and as genetic vaccines, an accurate assessment of the true incidence of latent adenoviral infections as well as the immune response to such infections is of paramount importance in furthering our understanding of adenoviral disease as well as to gauge the immune effects of future adenoviral vaccines such as those for HIV, tuberculosis, or malaria. We have recently determined that apes such as chimpanzees, gorillas and orangutans both in captivity and in the wild frequently shed live adenovirus in their stools [18]; in the same study, we also confirmed previous estimates [10], [19] where the adenoviruses could be detected in human stools relatively rarely, i.e., in about 2 to 3% of normal asymptomatic adults. In spite of this there is a high prevalence of CD4+ and CD8+ gut-associated lymphocytes that are sensitized to adenoviral antigens [20]. We have, therefore, undertaken studies to determine whether detection of adenoviruses in stools is an inadequate surrogate for the assessment of adenoviral residence in the gut and whether the prevalence of adenoviruses in the gut is in fact higher than is currently known.

## Methods

### Isolation of lymphocytes from human gut tissue

Surgically extracted ileum, colon and rectum specimens from human donors were provided by the Cooperative Human Tissue Network (CHTN) of the National Institutes of Health. Intestinal samples obtained at autopsy were obtained from the National Disease Research Interchange. All ethical procedures with respect to the protection of human-derived material required for access to CHTN supplied human tissues and summarized under Policies & Procedures for the Protection of Human Subjects (http://www.chtn.nci.nih.gov/access.html) were complied with. In all, intestinal tissue samples from 58 donors were received and analyzed for this study. To recover intra-epithelial lymphocytes (IELs), tissue samples were cut into 0.5-cm squares, washed, and incubated with continuous agitation in Hanks' balanced salt solution containing 5% fetal bovine serum (FBS) 0.75 mM EDTA, 100 U/ml penicillin, 100 µg/ml gentamicin, 25 mM HEPES buffer pH 7.4, and (HBSS-EDTA). The supernatant was removed and used for the isolation of IELs as described below. For the isolation of lymphocytes from the lamina propria (LPLs), tissue fragments were cut into 1- to 2-mm pieces and incubated with continuous agitation for 30 minute intervals in RPMI medium containing 0.5 mg/ml type II collagenase, penicillin, gentamicin, HEPES buffer, L-glutamine, and 5% FBS. At the end of each interval, intestinal pieces were further disrupted by agitating the pieces 15 times in a 20-ml syringe. The medium, containing LPLs, was separated from the remaining tissue fragments by passage through stainless steel screen cups (mesh size, 40 µm). This process was repeated two or three times, until the intestinal pieces had completely dissociated into small fragments. The lymphocyte population was enriched by discontinuous Percoll (GE Healthcare Biosciences) density gradient centrifugation which was performed by diluting isotonic Percoll to 35% (vol./vol.) and to 60% (vol./vol.) with RPMI and centrifuging the samples at 800×g for 20 min. LPLs were isolated from the interface between the 35% and 60% layers and re-suspended in complete RPMI medium. For the isolation of IELs, the supernatant from the HBSS-EDTA wash was transferred to a tube and cells washed in RPMI with 5% FBS and stored on ice. Fresh HBSS-EDTA was added to the intestinal pieces and the process was repeated at least twice till the supernatant was clear. The harvested cells present in the wash were pooled into one tube, filtered through a 40 µm sterile nylon strain, and further purified on a discontinuous Percoll gradient as described above.

### Fractionation of LPLs

Five to ten million LPLs isolated as described above were fractionated into CD4+ and CD8+ subsets using CD4 or CD8 Dynabeads® (Invitrogen) following the manufacturer's instructions. The CD4+-.and CD8+-depleted flow-through fractions were also collected.

### Isolation of DNA and RNA from lymphocytes isolated from gut tissue

Total cellular DNA and RNA were prepared from tissues or from LPLs or IELs using the Allprep DNA/RNA isolation kit purchased from Qiagen (Valencia, CA) following the manufacturer's instructions.

### Detection of adenoviral DNA

**Figure 1:**
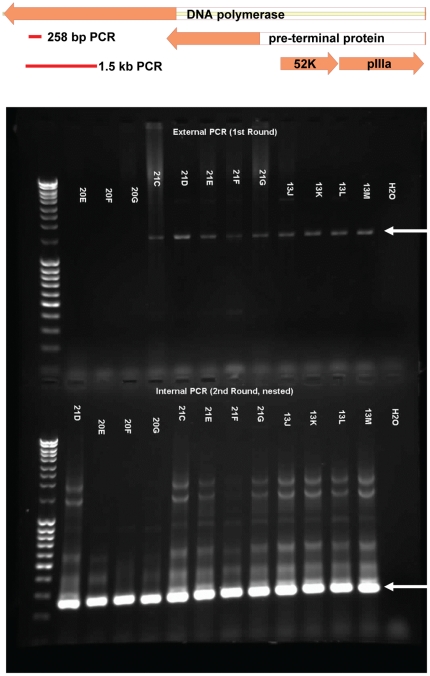
Detection of adenoviral DNA by nested PCR. Top – Diagram showing the region of the adenoviral DNA polymerase gene that was amplified. Primers designed to hybridize with sequences conserved across species A–F were used to amplify a 1.5 kb region. The product was used as template for a 2nd round of PCR using similarly conserved primers located internal to the first primer set that amplified a 258 bp product. Bottom – Agarose gel showing products of the 1st round PCR (upper gel panel) showing positivity (1.5 kb product) in some samples (arrow). The products of the first round were used for a 2nd nested PCR (lower gel panel).

Intestinal tissue fragments obtained from routine autopsies or surgical biopsy specimens were used in this study. DNA isolated from whole tissue fragments, as well as from lymphocytes isolated from them, was tested for the presence of adenoviral sequences by a nested PCR technique that amplified a 199 base pair portion of the adenoviral DNA polymerase gene. Nested PCR primers were designed to amplify adenoviruses from species A through G. The outer primers, TGATGCGYTTCTTACCTYTGGTYTCCATGAG and AGTTYTACATGCTGGGCTCTTACCG, amplify a 1.5 kb fragment of the adenoviral DNA polymerase gene (Figure 1); the products of this PCR reaction were used as template for a second round of PCR using the inner primers, GTGACAAAGAGGCTGTCCGTGTCCCCGTA and TCACGTGGCCTACACTTACAAGCCAATCAC, which amplifies a 258 bp fragment nested within the first product. In every case, the nested PCR products were cloned and sequenced to confirm the adenoviral origin of the amplified product. (Usually 2 clones were sequenced from each cloning experiment unless only one clone was obtained). The 199 bp sequence between the two primers was used for sequence comparisons.

### Quantitative PCR for the estimation of adenoviral DNA copies

Genomic DNAs isolated from CD4+ and CD8+ lymphocytes were subjected to real-time quantitative PCR analysis using the probe 6FAM-TGGTGGACAACAAGTC-MGBNFQ and the primers ACAGCATTCGTTACTCGG and CTGTGGTCGTTCTGGTAG which amplify a region of the penton-base gene that is highly conserved across adenovirus species, using HAdV-4 DNA as standard. The total genome copies input into the reaction was determined in parallel using the probe 6FAM-TCTTACCCTCAGCTGGAT-MGBNFQ and the primers AGGAACACTATAGCTCTCTGA and CTGCGCTCATCATGATGGA which estimates the number of copies of the genomic (X-linked) ornithine transcarbamylase gene. The determined adenoviral DNA copy numbers and the corresponding input genome copy number (adjusting as necessary for the sex of the subjects) were used to calculate adenoviral DNA copies per million diploid genomes.

### Reverse transcription PCR for the hexon (L3b) transcript

1 µg of total RNA was used for reverse transcription using the High Capacity cDNA Reverse Transcription kit from Applied Biosystems (Carlsbad, CA) in a total volume of 50 µl. Two microliters of the resulting cDNA was used in a PCR reaction using the primer ACTBTCTTCCGSATCGCTGT and CTCAGGTACTCCGAAGCATCCTG which amplifies an approximately 300 bp product comprised of the spliced tripartite leader and the beginning of the hexon coding region.

### Sequence analysis

Sequences were aligned using the AlignX module of Vector NTI software (Invitrogen Inc., Carlsbad, CA) utilizing a neighbor-joining algorithm of Saitou and Nei [21].

## Results

### Detection of adenoviral DNA in intestinal tissue and intestinal lymphocytes

Human intestinal tissues obtained during surgery or from autopsy were tested for the presence of adenoviral DNA using a nested PCR technique that amplifies a 199 bp segment of the adenoviral DNA polymerase gene (Figure 1). When the PCR reaction yielded a product of the correct size, it was cloned and sequenced. In all, total cellular DNA was extracted from the intestinal tissue from 58 individual donors which was used as template for detection of adenoviral DNA. Twenty-one of the samples were scored as positive based on the sequence of the amplified DNA. The species of adenovirus that was assigned based on the sequence is shown in Table 1.

**Table 1:** Detection of adenoviral DNA in intestinal tissue fragments.

Donor ID	Sex/Age	Ad. Species in tissue
1	F/91	E (1)
2	F/60	Negative
3	M/67	E (1)
4	F/53	Negative
5	M/75	E (1), E (4), E (7), E (8)
6	F/73	E (1), B (6)
7	F/63	E, B (6)
8		Negative
9	M/70	E (1)
10	M/68	Negative
11	M/70	Negative
13	M/50	B
14	M/18	Negative
16	F/71	Negative
17	F/35	Negative
18		Negative
20	F/56	Negative
21	F/71	Negative
22	M/42	E
23	F/68	E (2)
24	M/53	E (2)
25	M/57	E (2)
26	M/57	E (2)
27	F/61	Negative
28		E (2)
29	M/60	E (2)
30	M/49	Negative
31		Negative
70	F/57	Negative
71	F-64	Negative
72	F/58	Negative
73	F/56	Negative
74	F/49	Negative
75	F/50	Negative
76	F/78	Negative
77	F/25	Negative
78	F-49	Negative
79	M/57	Negative
80	M/31	F, F (9)
81	F/53	Negative
82	M/44	Negative
83	F/58	Negative
84	M/60	Negative
85	M/80	Negative
86	M/52	E, E
87	F/59	E
88	F/68	E
89	F/70	C, E (11)
90	F/39	Negative
91	M/60	Negative
92	M/32	E (1)
93	M/69	E (1)
94	M/56	Negative
95	F/68	Negative
96	M/60	Negative
97	F/60	Negative
98	F/61	Negative
99	M/51	Negative

**Figure 2:**
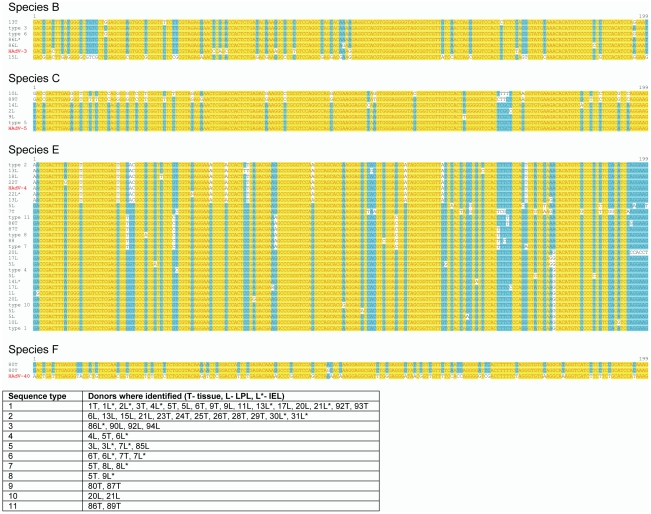
Alignments of the amplified 199 bp sequences contained internal to the primers of the 258 nested PCR products. The donor from whom each sequence was identified (as described in Tables 1 and 2) is indicated and corresponds to donor numbers in Tables 1 and 2. (The letter following the donor number identifies the source of the DNA used for analysis: T- intestinal tissue, L- LPLs, L*- IELs. All sequences were assigned to one of four adenoviral species, (HAdV-B, HAdV-C, HAdV-E, or HAdV-F) using the neighbor-joining algorithm of Saitou and Nei [21] and the resulting alignments with one of the reference HAdV species (red font) is shown. Regions of identity across all sequences are shaded yellow; regions that are common to the majority of sequences are shaded blue. Sequences that were obtained more than once were assigned to one of 11 “sequence types”. The donor sample that yielded each sequence type is shown in the table.

DNAs extracted from intestinal tissue from 58 donors were tested for the presence of adenoviral DNA by nested PCR. The sex (M/F) and age of the donor at the time of biopsy or autopsy is shown. When positive, the 199 bp sequence of the adenoviral DNA polymerase gene that was amplified was used to assign the species shown. The “sequence type” is shown in parenthesis after the assigned species in case of sequences isolated more than once and corresponds to the “sequence types” shown in Figure 2.

Because mucosal lymphocytes have long been implicated as the cell types that harbor latent adenovirus [6], [8], [22], [23], [24], a subset of the tissue fragments were used for the isolation of lymphocytes prior to DNA analysis. Lamina propria lymphocytes (LPLs) and intra-epithelial lymphocytes (IELs) were isolated as described in Methods. Total DNA was prepared from the isolated LPLs and IELs and subjected to nested PCR analysis for the detection of adenoviral DNA. When positive, the PCR products were cloned and sequenced and species assigned as before (Table 2). LPLs were found to be positive more often (21/24) than whole tissue, indicating that the sensitivity of detection by our nested PCR technique was higher when DNA from lymphocytes rather than the whole tissue was used as template.

**Table 2:** Detection of adenoviral DNA in intestinal lymphocytes.

Donor ID	Sex/Age	Adenovirus Species (sequence type)		
		Tissue	IELs	LPLs
1	F/91	E (1)	E (1)	E
2	F/60	Negative	E (1)	C
3	M/67	E (1)	C (5)	C (5)
4	F/53	Negative	E (1)	E (4)
5	M/75	E (1), E (4), E (7), E (8)	Not tested	E, E, E, E, E, E (1)
6	F/73	E (1), B (6)	E (4), B (6)	Negative
7	F/63	E, B (6)	C (5), B (6)	Negative
8		Negative	E (7)	E (7)
9	M/70	E (1)	E (8)	C, E (1)
10	M/68	Negative	Negative	C, E
11	M/70	Negative	Negative	E (1)
12	F/37	Not tested	Negative	Negative
13	M/50	B	E (1)	E, E, E, E (1), E (2)
14	M/18	Negative	E	C
15	F/18	Not tested	Not tested	B, E (2)
17	F/35	Negative	Negative	E, E, E (1)
18		Negative	Negative	E
20	F/56	Negative	Negative	E, E, E (1) E (10)
21	F/71	Negative	E (1)	E (2), E (10)
85	M/80	Negative	Not tested	C (5)
86	M/52	E, E	B (3)	B
90	F/39	Negative	Not tested	B (3)
92	M/32	E (1)	Not tested	B (3)
94	M/56	Negative	Not tested	B (3)

DNAs from intra-epithelial lymphocytes (IELs) and lamina propria lymphocytes (LPLs) from 24 donors were tested for the presence of adenoviral DNA by nested PCR. The sex (M/F) and age of the donor at the time of biopsy or autopsy is shown. When positive, the sequence of the adenoviral DNA polymerase gene that was amplified was used to assign the species shown (the result of the analysis of whole tissue DNA is also shown). All the identified sequences from any particular individual sample are indicated. The “sequence type” is shown in parentheses after the assigned species in case of sequences isolated more than once and corresponds to the “sequence types” shown in Figure 2.

As mentioned above, 58 total intestinal tissue samples were received and DNA was extracted from them. In the early part of our investigation, we found the prevalence of adenoviral DNA in total tissue DNA to be less than 50%, and this prompted us to test whether we may be able to improve the sensitivity of detection by first isolating lymphocytes – IELs and LPLs – from the intestinal tissues. However, we were not able to successfully isolate sufficient numbers of IELs/LPLs from every sample, probably relating to the large differences in sample quality with respect to factors such as the state of preservation following autopsy or biopsy, the amount of sample available, the location of the intestine resected etc., so that in all, lymphocytes from 24 donors could be analyzed. However when it became clear that a high proportion of gut lymphocytes we did analyze (particularly LPLs) were positive for adenoviral DNA, we additionally isolated RNA from the last 12 intestine samples that we received. Samples from which we were able to recover at least 1 µg of total RNA (11 donors) were subjected to RT-PCR for the presence of adenoviral transcripts, as described below.

In all, adenoviral DNA was successfully amplified from 39 different donors and 41 non-identical sequences were obtained. The sequences that were obtained were assigned to an adenoviral species by comparing the sequence with human adenovirus sequences (from species A, B, C, D, E, F and G) employing the neighbor joining algorithm of Saitou and Nei [21] using Vector NTI software. The alignments derived from this analysis are shown in Figure 2. Only one donor harbored sequences that were related to species F (HAdV-40 or 41), which can usually be causally related to infectious diarrhea [25]. HAdV-A or HAdV-D sequences have not been detected by us to date. A majority of the sequences clustered with HAdV-4, the only human species E adenovirus for which a full sequence in available. However it is interesting to note that only 6 of the sequences were clearly very closely related to the canonical species E (i.e., HAdV-4) sequence, and only one was identical. Alignments of the sequences that were assigned to belong to species B, C and E respectively are shown in Figure 2.

**Figure 3:**
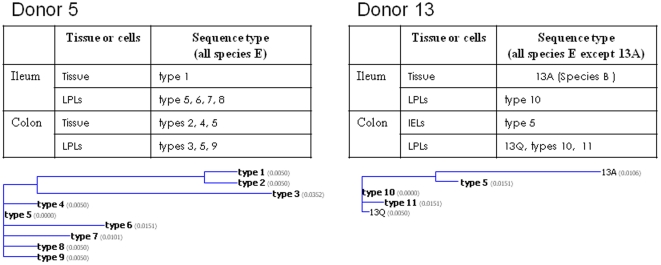
Two instances of multiple adenoviral DNA sequences amplified from a single donor. Sequences amplified from donors 5 and 13 and the source of the DNA from which sequences were amplified is shown in the tables. The phylogenetic relationships between these sequences as computed using the nearest neighbor algorithm of Saitou and Nei [21] is also shown. The calculated distance value for each sequence is shown in parentheses (Vector NTI, AlignX module).

It can be seen from the data shown on Table 1, Table 2 and Figure 2 that it was common to identify multiple sequences including those belonging to different species from the same donor. A large amount of tissue obtained at autopsy was made available to us from two donors (donors number 5 and 13). Donor 5 was a 75 year old white male who had died following a stroke. We performed nested PCR analysis on several tissue fragments from the ileum and colon as well as LPLs derived from those tissue fragments. We were able to identify nine non-identical sequences from this donor, all belonging to species E (Figure 3). Similarly donor 13 (a fifty year old white male who had died following myocardial infarction) yielded multiple sequences belonging to species E as well as one sequence that could be assigned to species B (Figure 3). Phylogenetic relationships of the sequences isolated from either donor are also shown in Figure 3.

### Detection and quantification of adenoviral DNA in fractionated lymphocytes

**Figure 4:**
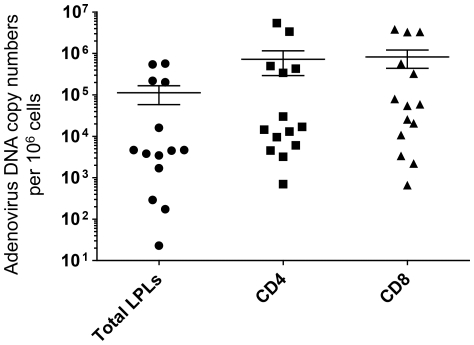
Copy numbers of adenovirus DNA (per million diploid genomes) detected by quantitative PCR in lamina propria lymphocyte (LPLs). Data for unfractionated (total LPLs) as well as CD4+ and CD8+ fractionated lymphocytes are shown. Each data point represents a single sample. The mean and standard deviation for each data set are indicated.

The cell type that has most often been shown to be a likely site for latent adenoviruses is the T lymphocyte [6], [8], [9], [22], [23], [24]. We wished to confirm that adenovirus DNA was present in the T lymphocyte fraction of the cells isolated from lamina propria and at the same time to test whether the adenoviral DNA was equally present in CD4+ and CD8+ cells. In order to do this, LPLs from 14 different subjects were fractionated into CD4+ and CD8+ subsets using antibody coated magnetic beads and total cellular DNA was extracted from them. A real-time PCR method was used to quantify the number of adenoviral DNA copies that were present in unfractionated lymphocytes (total LPLs) as well as the CD4+ and CD8+ fractions (Figure 4). The DNA copy numbers ranged from about a hundred to about half a million (per million cells) in the unfractionated LPLs; the fractionated lymphocytes harbored from a few hundred to as many as 3 million adenoviral DNA copies per million cells on average. In 12 of 14 samples, the copy numbers in the fractionated lymphocytes was higher than the copy numbers in the corresponding unfractionated samples (data not shown) indicating that the T lymphocytes present in the LPL population preferentially harbored adenoviral DNA; also, it was seen that adenoviral DNA was equally likely to be present in CD4+ or CD8+ lymphocytes.

### Detection of active adenoviral transcription

**Figure 5:**
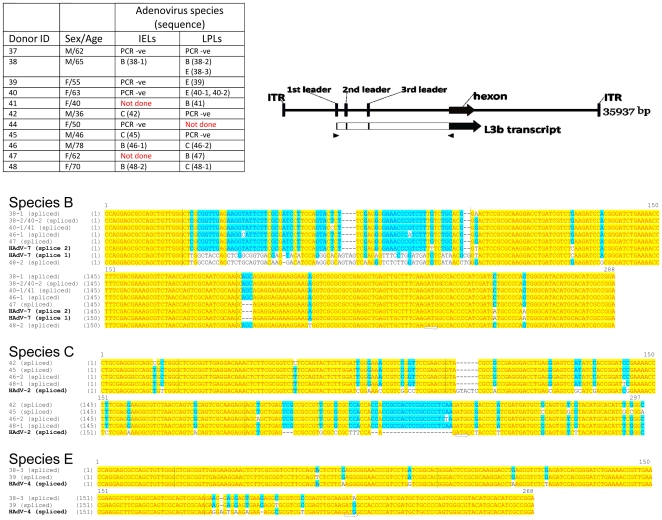
Detection of the adenoviral transcription. RNA was isolated from IELs and LPLs from 11 subjects (table, upper left) and subjected to reverse transcription followed by PCR. The adenoviral L3b transcript (shown in green in the diagram, upper right; hexon orf shown in orange) harbors a tri-partite leader comprised of three widely separated leader exons followed by the last exon encoding the hexon protein. The PCR primers (as shown by arrowheads) were designed to hybridize to conserved sequences in the first leader and to the last exon. The approximately 300 base-pair product from reactions that were positive were cloned and sequenced. The sequences obtained from the different samples were assigned to adenoviral species B, C, or E (table) and aligned to a reference adenovirus sequence (HAdV-7, HAdV-2, and HAdV-4, respectively) for each species. Regions of identity across all sequences are shaded yellow; regions that are common to the majority of sequences are shaded blue. The exon boundaries are indicated by vertical lines. The putative hexon start codon is indicated by an arrow below the alignments. One of the species B sequences (sequence 48-2) contains a second leader that represents an alternate splicing pattern from that seen with the other sequences.

Finally, we wished to determine whether the adenoviral DNAs that we could detect in the gut lymphocytes were in the process of being actively transcribed. LPLs and IELs were isolated from the intestinal tissue of 11 donors and total RNA was prepared from them. We attempted to detect the presence of the early region E1a transcripts using reverse transcription followed by PCR (RT-PCR). Because there were insufficient sequence identities in the E1a regions of different species of adenoviruses, we designed separate primer sets to detect E1a transcripts for species B, C, and E adenoviruses. However we were unable to detect E1 transcripts in any RNA sample, probably because transcript copy numbers were too low to be detected by our assay. Because transcripts from the adenoviral major late promoter are very abundant in replicating cells, we next designed primers to detect the spliced L3b transcript that encodes the hexon protein (Figure 5). The assay was designed to detect processed mRNA since the primers spanned introns and the PCR product corresponded to the size expected of fully processed mRNA. This also precluded the artefactual detection of adenoviral genomic DNA. We were successful in amplifying and sequencing the L3b late transcript in RNA from 9 of the 11 donors that were tested (Figure 5). Sequences that could be assigned to species B, C, and E could be identified; some samples yielded more than one kind of sequence, e.g., three different sequences could be identified in RNA from donor 38.

## Discussion

Persistent enteral infections with adenoviruses following resolution of upper respiratory tract infections (symptomatic or not) in infants and children have been well documented [1], [2]. Up to 15% of cases were found to excrete adenovirus in the stools for periods ranging from 3 months to a year following the initial infections; about a third of these continued to excrete virus for even longer periods. Furthermore many of these cases exhibited intermittent excretion confirmed to be due to recrudescence rather than re-infection [2], [26], implying periods of suppressed viral replication. While it is clear that adenoviral infections of the upper respiratory tract leads to fecal virus excretion, Adrian et al. [26] speculated that the location of the persistent infection could be the intestinal epithelium or Peyer's patches. In this communication we have directly tested intestinal tissue for the presence of adenoviral DNA and found that intestinal tissue, particularly lymphocytes isolated from intestinal tissues, can frequently be found to harbor adenoviral DNA. In contrast, DNA from respiratory inflammatory exudates, such as from patients with chronic obstructive pulmonary disease, are only infrequently positive for the presence of adenoviral sequences [27].

Adenoviruses have long been known establish persistent infections in adenoidal and tonsillar tissue as evidenced by successful outgrowth in culture [3], [5], [6], [9] or the presence of adenoviral DNA [7], [8]. When outgrowth in culture is successful, replication is seen to occur in fibroblasts or epithelial cells. However, the cell types that are now implicated to be the persistent carriers of adenoviruses in the tonsils and adenoids are lymphocytes where the adenoviral genome is presumed to be latent but capable of productive replication when stimulated with mitogens [9]. A similar re-activation is presumed to occur in immunocompromised states such as in transplant recipients where adenoviral viremia is a recognized complication [12], [13]. It has been recognized that although lymphocytic and macrophage cell lines can be infected with adenovirus, in contrast to epithelial cells or fibroblasts, a lytic infection often does not occur [23], [24], [28], [29], [30]. A persistent infectious state can be established in such cell lines with continuous low-level viral replication. Although we were able to detect late gene expression in most of the samples that we tested, we have so far been unable to recover live adenovirus from LPLs even after stimulating by various means; viz., with phytohemagglutinin, with phorbol myristate acetate in conjunction with ionomycin, or with a combination of anti-CD3 and anti-CD28 antibodies respectively (data not shown). This may be due to T cells being intrinsically resistant to viral replication as has been shown to be the case with human T cell lines [30]. It has been previously noted that even when adenoviral replication can be demonstrated in primary T cells from the adenoids or tonsils, they appear to be considerably attenuated in their capacity to initiate a typical cytopathic effect in culture [9]. It is possible that this is the case with adenoviruses from the gut as well.

In our quantitative PCR assays we determined the adenovirus copy numbers in gut lymphocytes ranged from a few hundred to several million per million cells. Similar copy number were reported for lymphocytes from adenoids and tonsils [9]; these investigators found that even in samples with very high adenovirus copy numbers, the actual numbers of cells harboring viral DNA (as determined by a limiting dilution assay) ranged from less than one in a million to one in a thousand. Although we have not determined this, we expect the same to be true for gut lymphocytes.

Although we have found that adenoviral DNA and adenoviral transcripts can easily be detected in intestine-derived lymphocytes, we and others have found that the frequency with which adenoviruses can be cultured from stools of normal human subjects is only about 2 to 3% [10], [18], [19] with the exception of subjects in recent contact with clinically ill adenovirus-infected patients, principally children, as first reported by Fox et al. [1], [2]. This is clearly different from the situation in both great apes and monkeys where shedding of live adenoviruses is easily detected [18]. Whether this reflects a difference in the immunological milieu between apes and monkeys on the one hand and humans on the other, or whether this is a consequence of the differences in the specific types of colonizing adenoviruses needs to be determined.

The frequent and facile demonstration of adenoviral DNA in lymphocytes of the adenoids and tonsils contrasted with the absence of such a demonstration in cells in systemic circulation raises the question whether this apparent compartmentalization of lymphocytes extends to other peripheral lymphoid organs. We now show that the T cells of the sub-mucosa of the ileum, rectum and colon are also colonized by adenoviruses which may be seen to be part of the normal flora. It would be important to test whether circulating PBMCs do in fact harbor adenoviral genomes less frequently than lymphocytes that home to the gut, preferably using samples from both locations in the same individual.

There appears to be a remarkable diversity of adenovirus sequences that can be recovered by PCR, including those belonging to species E that are different from the only readily isolated species E adenovirus – HAdV-4. The part of the adenovirus genome that was amplified and sequenced corresponds to the amino-acid residues 944–1009 of the HAdV-5 DNA polymerase, a 1198-residue protein. Over this segment the degree of intra-species sequence DNA sequence variation among sequenced human adenoviruses is very low. For instance there is 100% DNA sequence identity between the two sequenced species B1 human adenoviruses HAdV-3 and HAdV-7 and between the HAdV-B2 adenoviruses HAdV-11 and HAdV-35; only a 2-base difference is seen between the three HAdV-C adenoviruses (HAdV-1, HAdV-2, and HAdV-5). The DNA sequences that were identified in this study varied much more; for example among the five kinds of sequences identified that could be assigned to HAdV-B1 (13T, type 3, type 6, 86L* and 86L, Figure 2), we were able to detect 14 nucleotide differences from the reference HAdV-3 sequence (of which 8 were silent, data not shown). A similar degree of sequence diversity is also seen in the species C and species E sequences as well (Figure 2).

Although we have isolated a diverse array of sequences from human lymphocytes, we have also seen that it is common for a single individual to harbor multiple adenoviral sequences, including at least two individuals from whom we recovered multiple sequences as shown in Figure 3. However we have not systematically addressed the question of how common of an occurrence this may be, and whether these two individuals are more typical of the normal population than not.

We have also demonstrated that expression of adenoviral late genes is easily detected even though viral replication is likely to only occur at fairly low levels. We and others have shown that T cells with cross-reactivity across serotypes can be demonstrated in normal subjects [20], [31], [32]. The continuous low-level exposure to adenoviral antigens resulting from this smoldering viral infection may help explain the fairly widespread occurrence of T cells reactive to adenoviral antigens such as the hexon protein. Furthermore, the absence of florid viral replication may explain the generally low antibody titers that are normally seen in adult population [20], [33] compared to the titers that are observed following adenovirus infection [34], [35] or following adenovirus administration for immunization [36], [37]. It can be speculated that infected T cells should themselves be targets for cytotoxic cells; it would be of interest to determine whether adenoviral genes such as those expressed from early region E3 can influence this process and whether the expression profile of genes from the E3 region may be different in lymphoid cells than in somatic cells.
